# Increased healthcare costs in COVID-19 patients with unhealthy habits: The case of smoking

**DOI:** 10.18332/tid/163301

**Published:** 2023-06-19

**Authors:** Eva María Andrés Esteban, Alfredo Cabezas Ares, María Jesús Delgado Rodríguez

**Affiliations:** 1 Department of Business Economics (ADO), Applied Economics II and Fundamentals of Economic Analysis, Universidad Rey Juan Carlos, Madrid, Spain

**Keywords:** tobacco, health cost, smoking prognosis, COVID-19, economic burden

## Abstract

**INTRODUCTION:**

This study aims to analyze the differences in the prognosis and cost of COVID-19 patients in terms of mortality and occurrence of complications due to tobacco use.

**METHODS:**

This study was conducted using a unique Spanish electronic database built by health professionals during the first wave of the pandemic on the admission and evolution of a patient infected by the SARS-CoV-2 virus. Data were collected on all patients admitted to La Paz hospital (Madrid) from the pandemic’s beginning until 15 July 2020. Demographic factors and the incidence of complications in smoker and non-smoker patients were compared using the Mann-Whitney U-test or chi-squared test. Survival analysis was performed using the Kaplan-Meier estimator and Cox regression. Finally, the costs between the two groups were estimated using a Generalized Linear Model.

**RESULTS:**

A total of 3521 patients were included in the analysis, with median age of 62 years (IQR: 47–78), 51.09% were women, and 16.42% were smokers. Patients who smoked had a higher incidence of complications during their hospital stay, especially complications related to the respiratory and cardiac systems. They were also associated with a worse prognosis in terms of the need for ICU admission and mortality, leading to an increase in the management cost of the smoking COVID-19 patients by 14.72%.

**CONCLUSIONS:**

Healthcare in Spain is mainly financed by the national tax system, so introducing an additional financing system for pathologies related to the consumption of addictive substances and associated diseases and complications would decrease the burden on the economy in terms of healthcare.

## INTRODUCTION

The 2019 SARS-CoV-2 virus infection disease pandemic, known as COVID-19, was born as an infection caused by a severe acute respiratory syndrome coronavirus and has now resulted in more than 665 million cases worldwide and more than 6.7 million deaths, according to the Johns Hopkins University’s independent tally^1^, which monitors the coronavirus situation with comprehensive data from all countries since the beginning of the pandemic.

Tobacco smoking is a known significant risk factor for chronic and severe diseases and even for a worse prognosis in many respiratory infectious diseases^2^. This suggests that COVID-19 patients may have further complications. However, the effects of smoking on COVID-19 need further analysis. Some epidemiological studies on smoking and SARS-CoV-2 infection suggest that active smoking is associated with increased severity of illness and death in hospitalized patients with COVID-19^3,4^. However, not all studies reach this conclusion during the pandemic^5^, despite the attempts made to analyze whether smoking is associated with an increased risk of respiratory infection^6^ or a higher incidence of complications during hospitalisation^7,8^. In this regard, several meta-analyses presented on the effect of smoking on the severity of COVID-19^9-11^ state that smokers have a higher risk of admission to the hospital with a severe clinical presentation and are also approximately twice as likely to experience severe or critical COVID-19 compared to ex-smokers or never smokers.

All this suggests that the cost of managing smoking patients is much higher than non-smoking patients, as they arrive at the emergency department with a worse clinical picture and are also associated with a higher incidence of complications. However, no studies show the cost of management of COVID-19 patients due to the heterogeneity of prices. Still, Calderón-Moreno et al.^12^ estimate a cost of €4294.36 in hospitalized patients who have not required admission to intensive care units and €14440.68 in patients admitted to the ICU. Our working hypothesis is that smokers have an increased average cost relative to non-smokers, mainly due to the occurrence of respiratory or cardiac complications and a worse prognosis in terms of mortality or need for ICU admission.

## METHODS

### Population and sample

The La Paz electronic data collection notebook (CRDe) is a database created at the Hospital Clínico Universitario La Paz where sociodemographic, clinical, analytical and management information is collected on patients admitted for respiratory infection by SARS-CoV-2 coronavirus from the beginning of the pandemic until 15 July 2020. This study extracts information from 3581 patients of legal age admitted during the study period with a diagnosis of COVID-19. The study was conducted following the principles of the Declaration of Helsinki (2008 update; available on the World Medical Association website - https://www.wma.net/policies-post/wma-declaration-of-helsinki-ethical-principles-for-medical-research-involving-human-subjects/) and under the standards of good clinical practice described in the ICH Tripartite Harmonized Guidelines for Good Clinical Practice (1996), and the guidelines for Good Epidemiological Practice (https://www.ema.europa.eu/en/ich-e6-r2-good-clinical-practice-scientific-guideline). The study was approved by the Clinical Research Ethics Committee of the Hospital Universitario La Paz (Madrid).

For the study characteristics and evolution depending on smoking status, all 3521 patients’ information was analyzed. For the economic study of the healthcare cost of COVID-19 patients, deceased patients were eliminated before analysis as this could lead to artificially underestimating or overestimating the average costs of patient management. Furthermore, it would be challenging to describe the treatment, management or incidence of complications for any of them. Finally, costs related to deaths are always difficult to attribute to a specific disease, and the economic burden of end-of-life is often considered separate from the burden of disease.

### Variables

Information is collected on demographic characteristics, history and comorbidities, clinical variables and variables related to patient prognosis (total days of admission, days of stay in ICU or exitus). The research has considered the severity of the patient on admission to the hospital using the Confusion, Urea, Respiratory Rate, Blood Pressure and Age (CURB-65) score, the Pneumonia Severity Index (PSI), the Sequential Organ Failure Assessment (SOFA) and the quick Sequential Organ Failure Assessment (qSOFA), and the scale of the Pneumonia Outcomes Research Team (FINE). The daily costs of hospitalization on the ward, in the ICU, and the management of complications of COVID-19 patients, were obtained from the accounting department of the Hospital Universitario La Paz.

### Statistical analysis

Quantitative variables were described using median and interquartile range and qualitative variables by frequency and percentage. For the comparison of variables between smokers and non-smokers, the non-parametric Mann-Whitney U test was used for quantitative variables, as the distribution of all variables did not assume normality, using the Shapiro-Wilks test for normality. For comparison with qualitative variables, the chi-squared test was used.

Survival estimates were made using the Kaplan-Meier method, comparing the survival curve between smokers and non-smokers using the Wilcoxon test because the curves did not reach the median survival. Multivariate analysis was performed by Cox regression, using the forward conditional method, introducing variables that were significant in the bivariate analysis as independent variables. The multivariate model results are presented as hazard ratio (95% CI).

### Economic analysis

An analysis of the total care consumption of COVID-19 patients was conducted using data collected in a collective perspective limited to direct costs. For each patient, the cost included pharmacological treatments, healthcare personnel costs, analytical costs, imaging tests (mainly X-rays and MRI), hospital stay, and ICU stay. These costs were obtained from the accounting department of the Hospital La Paz (Madrid). On the other hand, the costs of complication management were assessed based on the 2020 National Costs by DRG survey. A cost comparison was made between smokers and non-smokers, and multivariate analysis was performed using a linear regression model to assess differences in patient profiles by patient management and the incidence of complications and to estimate total healthcare costs after adjusting for smoking. A sensitivity cost analysis was performed with patients who died during hospitalization, dividing this sample into smokers and non-smokers. All analyses were performed using STATA/SE v.16.0, and p<0.05 was considered significant.

## RESULTS

### Characteristics of the study population

Complete information on all variables was collected for 3521 patients, 48.91% of whom were male and 16.42% were smokers. Table 1 describes the demographic characteristics and clinical variables of the studied patients. The median age was 62 years (IQR: 47–78), and 19.80% were healthcare workers; 17.09% of the patients could have been infected by contact with a positive COVID-19 and 30.56% by nosocomial transmission.

**Table 1:** Demographic characteristics, severity scales and clinical variables in smokers and non-smokers among the studied patients

*Variables*	*All n (%)*	*Non-smokers n (%)*	*Smokers n (%)*	*p*
**Gender**				<0.001
Male	1722 (48.91)	1331 (45.23)	391 (67.65)	
Female	1799 (51.09)	1612 (54.77)	187 (32.35)	
**Age** (years), median (IQR)	62 (47–78)	60 (45–78)	68 (56–78)	<0.001
**Health worker**	667 (19.80)	582 (20.70)	85 (15.15)	0.003
**Housing conditions**				0.104
Not known	3162 (90.60)	2640 (90.44)	522 (91.42)	
Residence	314 (8.99)	270 (9.25)	44 (7.71)	
Hostel	13 (0.37)	8 (0.27)	5 (0.88)	
Prison	1 (0.03)	1 (0.03)	0 (0.00)	
**Relationship with COVID-19 positive**	554 (17.09)	471 (17.32)	83 (15.96)	0.452
**Suspected nosocomial transmission**	1064 (30.56)	894 (30.75)	170 (29.62)	0.589
**Functional stage** ^a^				0.101
Dependent for basic activities of daily living	252 (7.44)	222 (7.86)	30 (5.36)	
Semi-dependent for basic activities of daily living	190 (5.61)	155 (5.49)	35 (6.25)	
Independent for basic activities of daily living	2942 (86.94)	2447 (86.65)	495 (88.39)	
**Charlson comorbidity index, median (IQR)** ^b^	2 (0–5)	2 (0–4)	4 (2–6)	<0.001
**Severity scales**, mean ± SD^c^				
CURB-65	1.06 ± 1.14	1.02 ± 1.14	1.27 ± 1.15	<0.001
FINE	2.19 ± 1.59	2.06 ± 1.52	2.88 ± 1.86	0.084
qSOFA	0.33 ± 0.61	0.32 ± 0.60	0.38 ± 0.70	0.081
SOFA	0.57 ± 1.74	0.64 ± 1.93	0.27 ± 0.46	0.892
PSI	4.24 ± 14.75	4.85 ± 15.88	0.63 ± 1.77	0.015
**Need for oxygen therapy on admission**	2178 (63.22)	1176 (61.80)	402 (70.53)	<0.001
**Type of oxygen therapy**				<0.001
Venturi mask	178 (8.18)	144 (8.12)	34 (8.46)	
Single mask	14 (0.64)	12 (0.68)	2 (0.50)	
Nasal goggles	1170 (53.79)	994 (56.06)	176 (43.78)	
Mask with reservoir	567 (26.07)	442 (24.93)	125 (31.09)	
**Non-invasive mechanical ventilation**	109 (5.01)	74 (4.17)	35 (8.71)	
**Invasive mechanical ventilation**	137 (6.30)	107 (6.03)	30 (7.46)	
**Use of pronation technique and prone position**	188 (6.43)	137 (5.63)	51 (10.41)	<0.001
**Respiratory rate**, median (IQR)	18 (18–20)	18 (18–18)	18 (18–30)	0.002
**Need for admission to ICU**	173 (5.08)	138 (4.86)	35 (6.22)	0.180

a Functional stage of a patient refers to the dependency to perform basic daily activities.

b The Charlson comorbidity index is used for assessing life expectancy at ten years, depending on the age at which it is assessed and the subject’s comorbidities. It is widely used in clinical studies to adjust for poor patient prognosis.

c Severity scales measured on admission. IQR: interquartile range.

ICU: intensive care unit.

On arrival at the emergency department, the need for oxygen therapy was 63.22%, with nasal goggles being the most common way of providing oxygen to the patient. Only 5.08% required admission to the ICU on arrival at the hospital emergency department.

Regarding the differences between smokers and non-smokers, the group of smokers showed greater severity on admission to the hospital on the CURB-65 and PSI scales. This group also required greater respiratory assistance through oxygen therapy on admission, the use of prognostics and a greater need for mechanical ventilation, as shown in Table 1.

### Incidence of complications in patients admitted for COVID-19

To analyze the prognosis, Table 2 shows how the occurrence of complications related to the circulatory system, such as heart failure, arrhythmias or cardiac arrest, as well as coagulopathies, all of which are more common in patients who smoke. Likewise, neither hospital nor ICU stay was statistically different between smoker and non-smoker patients, but mortality was, with smokers reaching a mortality rate of 27.48% versus 17.34% (p<0.001).

**Table 2:** Incidence of complications during hospital admission for COVID-19 in smokers and non-smokers

*Complications*	*Non-smokers n (%)*	*Smokers n (%)*	*p*
Infections on admission	215 (7.48)	55 (9.58)	0.088
Specified micro-organism	128 (4.53)	29 (5.10)	0.554
Bacterial pneumonia	124 (4.33)	25 (4.36)	0.971
Acute respiratory distress syndrome	186 (6.49)	45 (7.87)	0.230
Pneumothorax	16 (0.56)	4 (0.70)	0.689
Pleural effusion	36 (1.26)	12 (2.09)	0.119
Seizures	10 (0.35)	2 (0.35)	1.000
Stroke	16 (0.56)	3 (0.52)	0.916
Congestive heart failure	59 (2.06)	25 (4.36)	0.001
Myocarditis	8 (0.28)	1 (0.17)	0.654
Pericarditis	2 (0.07)	1 (0.17)	0.438
Endocarditis	1 (0.03)	0 (0.00)	0.655
Arrhythmia	66 (2.31)	24 (4.19)	0.049
Cardiac ischemia	4 (0.14)	1 (0.18)	0.838
Cardiac arrest	38 (1.33)	14 (2.44)	0.047
Bacteremia	49 (1.71)	13 (2.28)	0.348
Alteration of coagulation	108 (3.77)	33 (5.76)	0.029
Subsidiary anemia	55 (1.92)	14 (2.45)	0.413
Rhabdomyolysis	7 (0.24)	4 (0.70)	0.080
Acute renal failure	239 (8.35)	64 (11.19)	0.029
Gastrointestinal bleeding	20 (0.70)	7 (1.22)	0.196
Pancreatitis	0 (0.00)	1 (0.17)	0.025
Acute confusional syndrome	252 (8.82)	64 (11.17)	0.076
Psychiatric complications	49 (1.71)	9 (1.57)	0.808
Adverse reaction	77 (2.69)	23 (4.01)	0.086
Exitus	487 (17.34)	155 (27.48)	<0.001
ICU stay (days), median (IQR)	10 (4–18)	7 (3–15)	0.208
Hospital stay (days), median (IQR)	14 (8–19)	14 (8–21)	0.112

**Figure 1:**
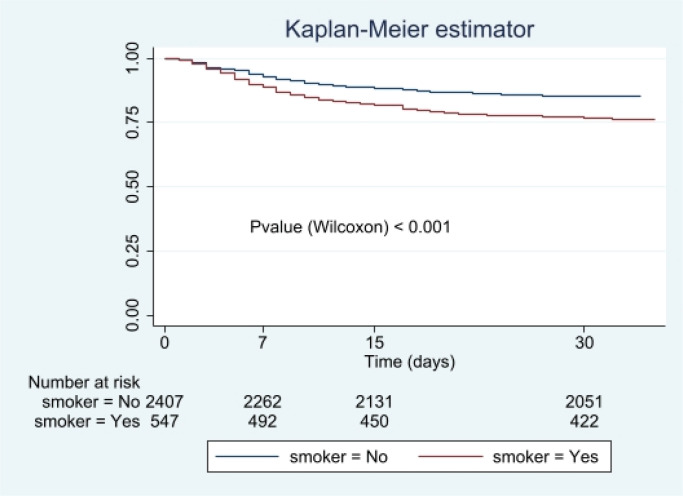
Kaplan-Meier survival estimator in smoking and non-smoking COVID-19 patients (the y-axis indicates cumulative survival probability)

The Kaplan-Meier estimator for survival analysis (Figure 1) shows that smokers die earlier than non-smokers, and the difference is statistically significant (p<0.001). Multivariate analysis (Table 3) showed that mortality is associated with smoking, with a hazard ration (HR) of 1.28 (95% CI: 1.06–1.54), indicating that a smoker has a 28% higher risk of dying from COVID-19 than a non-smoker. Other factors related to mortality, such as chronic heart disease (HR=2.00; 95% CI: 1.67–2.39), diabetes (HR=1.37; 95% CI: 1.14–1.65) or dyslipidemia (HR=1.32; 95% CI: 1.11–1.58) could be indirectly related to smoking.

**Table 3:** Cox regression model to predict COVID-19 mortality

	*HR (95% CI)*	*p*
Smoker	1.28 (1.06–1.54)	0.012
Hypertension	2.05 (1.68–2.49)	0.000
Chronic cardiac disease	2.00 (1.67–2.39)	0.000
Diabetes	1.37 (1.14–1.65)	0.001
Dyslipidemia	1.32 (1.11–1.58)	0.002
Chronic kidney disease	1.87 (1.52–2.31)	0.000

HR: hazard ratio.

### Economic analysis

Table 4 shows the cost during admission of a smoker hospitalized for COVID-19 versus a non-smoker. It is in the management of complications where there is the greatest difference in the management cost of the smoking patient (2218.34 vs 1622.10 €, which is a difference of 36.76%), as a smoking patient has a higher incidence of complications during the hospital stay. On the other hand, there is no major difference in the cost of hospital stay or intensive care units (626.50 vs 636.08 €), although there is a difference in the cost of imaging tests, both radiography and CT scans, as can be seen.

**Table 4:** Estimated cost* in smoking and non-smoking patients during the first wave of COVID-19

	*Hospitalization*	*ICU*	*Management of complications*	*X-ray costs*	*MRI costs*	*Total direct costs*
**Costs** (excluding fatalities)						
Smokers (N=423) (€)	4328.33	672.50	2218.34	30.80	178.76	7428.73
Non-smokers (N=2456) (€)	4036.07	636.08	1622.10	25.76	155.28	6475.29
Difference (€)	292.26	36.42	596.24	5.04	23.48	953.44
Cost increase (%)	7.24	5.73	36.76	19.57	15.12	14.72
Total smoking-adjusted direct cost estimate (€)		6615.38 (95% CI: 5008.18–8222.58)				
**Costs** (only fatalities)						
Smokers (N=155) (€)	2345.12	298.75	927.45	24.87	93.48	3689.67
Non-smokers (N=487) (€)	2537.89	345.12	918.33	19.45	80.19	3900.98
Difference (€)	-192.77	-46.37	9.12	5.42	13.29	-211.31
Cost increase (%)	-7.60	-13.44	0.99	27.87	16.57	-5.42

* The cost data presented in this table refer to the average cost per patient.

The cost increase for a smoking COVID-19 patient amounts to 14.72% or equivalently €953.44 per person. The item that most reflects the increase is the management of complications due to the high incidence (36.76%) of respiratory and cardiac complications, which similarly leads to a higher cost in radiological tests, 19.57% for radiography and 15.12% for computed tomography (CT).

The total cost of illness is estimated using a linear regression model, introducing as a dependent variable the direct cost of patient management COVID-19 adjusted for smoking, giving a total cost of 6615.38 € (95% CI: 5008.18–8222.58).

To complete the analysis, a sensitivity study was carried out. The sensitivity analysis shows the analysis of patients who died during the first wave (Table 4). The costs of patient management were lower in terms of length of stay and decreased in non-smokers by 7.60% and 13.44% in smokers. The cost of disease management was very similar in both groups.

## DISCUSSION

The COVID-19 pandemic is proving to be not only a health but also an economic and social challenge worldwide^13^. China isolated the city of Wuhan on 23 January 2020. From that date onwards, more than a third of the countries followed the same isolation policies imposed by the governments, with the main objective of reducing the spread of the disease among the population and thus avoiding the collapse of the health system^14^. These measures produced a worldwide economic setback, mainly due to redundancies, job cuts, increased health costs, social policies to alleviate the lack of income of many families affected by the pandemic, etc. Some figures show that the economic slowdown alone pushed more than 420 million people into absolute poverty, defined as an income below $1.90 per day^15^.

The economic cost of the disease caused by the SARS-CoV-2 virus has been little studied in the scientific literature. In Spain, we only found references to the healthcare cost of the disease in patients with COVID-19 disease, regardless of whether they have had to be hospitalized or not, with the figures amounting to €729.79 if the patient is treated only by the family doctor and between €4294.36 and €14440.68 if they are hospitalized depending on the severity and need for patient care^12^. Our estimates align with those described in Calderón-Moreno et al.^12^, with an average expenditure per patient of €6475.29 in non-smoking hospitalized patients and an increase of 14.72% in the case of an active smoker.

Our sensitivity analysis shows that the costs for patients who died during the first wave of COVID-19 were lower than those who survived. This may be due to several reasons, the main one being the lack of resources during that period, which meant that patients with a poor prognosis lacked adequate care for their clinical symptomatology. Spanish hospitals during this wave assigned preferences to patients according to their probability of survival. All this meant that the cost of managing COVID-19 patients who died was lower, as their hospital stay was much shorter than that of patients who survived, and the allocation of resources was more limited.

Smoking has not always been associated with higher healthcare costs. Tiihonen et al.^16^ stated that smoking is associated with a higher mortality rate and, therefore, with a moderate decrease in healthcare costs and a decrease in pension costs due to increased mortality. Our results show that smokers have a higher mortality rate and a more severe clinical picture in the case of hospitalization for SARS-CoV-2. This fact is observed in the hospital or ICU stay, where we did not find significant differences in the length of hospitalization of patients despite the difference in the severity of the symptomatology. This is because COVID-19 patients who smoke have a higher hospital mortality rate. Given their severity and the appearance of respiratory and cardiac complications, the cost of this specific diagnosis is €924.92 higher in this group if we take into account those who died and €596.24 if we do not take into account the patients who died during their stay in the hospital, highlighting the worse prognosis associated with smoking.

In the same study, they also state that, when the monetary value of years of life lost was considered, the net beneficial effect of not smoking for society was about €70000 per individual, and we think this may be the case for COVID-19 patients. In the case of the UK^17^, smoking remains a considerable public health burden. Therefore, the government prioritizes analyzing the burden in terms of mortality, disability, and economic costs to establish guidelines for national public health policy intervention. In Spain, the national health system is mainly financed by the national tax system, so introducing an additional financing system for addiction-related pathologies with addiction-related diseases and complications would decrease the burden on the economy in terms of public health.

### Strengths and limitations

Our study is supported by an extensive database created in March 2020 with the sole objective of studying the disease developed by the SARS-CoV-2 virus, so for the cost study, the COVID-19-related complications associated with this first wave, the most aggressive of the six recorded in Spain, are known. Also, all previous comorbidities could have been related to a worse prognosis, any pharmacological treatment, and imaging or analytical tests performed in these patients. As the data were not collected from the clinical history, the completion of this data collection notebook was carried out by dozens of healthcare professionals hired exclusively for this purpose, so the degree of completion and the quality of the information obtained is excellent. This database contains a total of 3581 patients, which means that the results obtained in this study are compelling and in line with the reality of what happened during this period.

As a limitation, we can point out that we only have information on the first wave, and we currently know that the virulence of the disease and the rate of infection varied over the following waves with the different mutations of the SARS-CoV-2 virus. As a consequence of this limitation, we believe that the costs of COVID-19 patient management varied in the different waves, but we cannot quantify these with data.

## CONCLUSIONS

Smoking patients not only have a worse outcome in the event of SARS-CoV-2 infection, but the cost of patient management is much higher than that of a non-smoking patients. This makes this a very important topic for society since COVID-19 has significantly increased healthcare costs that governments must manage. In countries such as Spain, where public healthcare is free and universal, the debate on increasing public resources to address rising costs is crucial. Policies should be created to raise public awareness of the economic impact of smoking on the management of any disease, including COVID-19. So far, in Spain, smokers are financing the increased cost they generate with excise taxes. Still, these taxes will have to be evaluated to see if they are sufficient to finance this increase in expenses derived from the impact of COVID-19 on smokers.

## Data Availability

The data supporting this research are available from the authors on reasonable request.
